# On Determining the Age Distribution of COVID-19 Pandemic

**DOI:** 10.3389/fpubh.2020.00202

**Published:** 2020-05-15

**Authors:** Dominic Cortis

**Affiliations:** Department of Insurance, FEMA, University of Malta, Msida, Malta

**Keywords:** crude death rates, COVID-19, age distribution, South Korea, China, 2019 pandemic, pandemic, coronavirus

## Abstract

Pandemics tend to have higher occurrence (morbidity) in younger individuals but higher mortality for the elderly. The higher rate of mortality of COVID-19 in elderly individuals has been discussed in many reports. However, this pandemic is a double-edged sword as this comment shows higher morbidity rates in elderly as well. This is shown by comparing the age distribution of cases in China and South Korea to the relative populations. In every case, the relative number of elderly contracting the virus is far higher than the proportion of elderly in the population. This is unlike past pandemics and shows that aging populations are at an even higher risk than the perceived age dependent rates may imply.

The crude death rate for COVID-19 cases reports has ranged significantly. For example, Guan et al. (1) reports a death rate of 1.4% while Baud et al. (2) reports 5.7%. The largest study published to date (44,672 cases) reports a death rate of 2.3% (3).

There are a considerable number of factors that affect the crude rate. The numerator can be affected by the fact that any current statistics would be based on the number of deaths to date as a fraction to the number of confirmed cases—with some of the latter ending in death at a later stage (2).

The denominator can be affected by the number of tests conducted and the age distribution. A higher number of tests conducted is likely to include a higher proportion of asymptomatic people or people with mild symptoms that may have been missed, hence lowering the crude rate.

A higher strain on the health system may lead to a lower proportion of asymptomatic people or people with mild to moderate symptoms being tested as well as a higher proportion of deaths on clinical cases due to lack of resources, affecting both the numerator and denominator.

These factors are inter-related. For example, younger individuals tend to have a higher proportion of asymptomatic people or people with mild symptoms who are likely to be missing from any statistics in extreme scenarios. Moreover, the final crude rate is not only dependent on the age specific mortality rates but also the distribution of ages for COVID-19 positive cases.

This comment discusses the distribution of confirmed COVID-19 cases in relation to the population distribution for three studies.

## Methodology

The three studies selected are Zhang (3), Guan et al. (1), and Korea Centers for Disease Control and Prevention (4). The first two are based in China at a point were most COVID-19 cases would have emerged and be concluded by now. The last report would be able to provide a comparison on the age distribution for cases in the country with one of the highest proportion of tests performed per million people^1^.

National Office statistics tend to group age distribution in three cohorts: less than 15 years old (youths), 15 to 64 years old (working population), and above 65 years old (elderly). The distribution of expected cases based on standard population statistics is compared to the same distribution for observed COVID-19 cases together with the old age dependency ratio (OADR) being the ratio of elderly to working population for each study.

Korean national office statistics (5) also show an additional grouping (being 3.2% of total) marked as foreigners. For simplication, 80% of foreigners are set in the working population cohort and 10% are set in each of the other two cohorts.

Zhang (3) and Korean Centers for Disease Control and Prevention (4) report COVID-19 positive cases and deaths in 10-year ranges. The age ranges 10–20 and 60–70 are assumed to be uniformly distributed and hence half of the frequencies for each (10–20 and 60–70) are included in the working population cohort.

Zhang (3) includes 33,367 cases based in Hubei province and 11,305 cases based in the rest of China. The expected age distribution is weighted to this ratio using the values from the China Statistical Yearbook (6). Guan et al. (1) investigates 1,099 cases spread over 30 provinces. A similar weighting based on the number of cases sampled by Guan et al. (1) per province is applied to produce the expected standard distribution. The two estimated age distributions for China are fairly similar with the one for Zhang (3) more heavily weighted on Hubei's statistics that has a slightly more aging population (OADR of 17.00%).

## Results

The table below summarizes the results obtained comparing the distribution of ages for the three major cohorts. The percentage of youths with confirmed COVID-19 cases is far lower than the standard population percentage, even in South Korea where a larger proportion of tests were held. The proportion of COVID-19 confirmed cases for youths is lower in China (1.55%, 0.89%) than South Korea (4.04%) as individuals with mild symptoms would have not been tested as in South Korea.

**Table:** 

		**0–14 years**	**15–64 years**	**65 years +**	**OADR**
Zhang (*n* = 44,672)	Estimated	16.08%	71.91%	12.01%	16.70%
	Actual	1.55%	76.93%	21.53%	27.99%
Guan (*n* = 1,099)	Estimated	16.36%	71.89%	11.75%	16.35%
	Actual	0.89%	83.98%	15.13%	18.02%
KCDC (*n* = 9,661)	Estimated	12.87%	72.49%	14.64%	20.20%
	Actual	4.04%	78.60%	17.36%	22.09%

The reduction in youths with clinically apparent COVID-19 cases does not result in a proportional increase for all other age groups but is more weighted to older individuals. This is shown by a higher old age dependency ratio for the actual cases in every scenario. One must consider that for the scenario generated by Guan et al. (1) the ratio of older individuals may have only increased 3.38% in absolute terms but this is a 28.77% growth in relative terms.

## Limitations

This is a brief report set in a scenario that is updating on a daily basis. The statistics used may not be complete. For example, Wuhan has recently revised its COVID-19 death toll upwards by 50% (7) and mortality statistics across many countries show excess number of deaths than reported (8, 9).

This leads to many limitations, including the robustness of the results. The aim of this comment is to generate ideas around future possible research and early indications of risk.

Another limitation is that countries, or even regions within the same country, may have had different approaches. In a stressed scenario, the public healthcare system would only be recording extreme cases, which tend to be elderly individuals with respect to COVID-19. That may mean that the skewness toward elderly may be biased. However, it also proves that, if there is a skewness, it leans toward having COVID-19 manifest itself relatively more in elderly.

## Discussion

These observations add to the ongoing discussion that the virus is highly contagious for elderly individuals, not only due to a higher rate of mortality^2^, but also due a higher proportion of cases. In essence, aging populations may be at increased risk from a 2-fold effect. If a population has a higher proportion of elderly, the proportion of confirmed COVID-19 cases would be higher, accentuated further if no normal tests are made. This is substantially different than what is typically reported for influenza (10) or other pandemics (11) which tend to have higher morbidity for younger individuals. For example, Lemaitre and Carrat (12) show that the relative ratios of morbidities were much higher for younger individuals than older ones in USA and France for the pandemics in the late 1970s (H1N1), late 1980s (H3N2) and in 2009 (H1N1).

It is therefore ideal that age specific mortality rates together with age specific count of cases are reported rather than crude rates and total counts of positive cases.

The three studies sampled have an estimated OADR of lower than 21%. In each of the three scenarios above, the relative growth in the elderly cohort ranges from 18.56% (KCDC) to 79.27% (Zhang) in relative terms.

The OADR in Korea and China is lower than any European country except for North Macedonia (20.2%), Andorra (18.7%), Armenia (17.6%), Turkey (12.9%), and Azerbaijan (9.6%). The European Union area has an average old-age dependency ratio of 31.0% while Italy has the highest rate at 35.7% (13). Therefore, the frequency of the pandemic in Italy can be partially described by its relatively older population. Other countries at increased risk due to high OADR are Japan, Finland, Portugal Greece, Germany Bulgaria, France, and Sweden (14). Age distributions can also partially explain why some countries such as Turkey have a low COVID-19 mortality rate despite the high number of cases.

As some countries are at different stages of the pandemic, further evaluation of the age distribution by morbidity would be of interest to prepare for future strains of COVID-19 or a possible second wave.

## Author Contributions

The author confirms being the sole contributor of this work and has approved it for publication.

## Conflict of Interest

The author declares that the research was conducted in the absence of any commercial or financial relationships that could be construed as a potential conflict of interest. The reviewer MF declared a past co-authorship with the author DC.

## Footnotes

^1^https://ourworldindata.org/covid-testing

^2^For example, no reported cases of death in Korean Centers for Disease Control and Prevention (4) and Zhang (3) for anyone under the age of 10.

## References

[1] Guan WJ Ni ZY Hu Y Liang WH Ou CQ He JX Clinical characteristics of coronavirus disease 2019 in China. New Engl J Med. (2020) 18:1708–1720. 10.1056/NEJMoa2002032 PMC7092819 32109013

[2] Baud D Qi X Nielsen-Saines K Musso D Pomar L Favre G. Real estimates of mortality following COVID-19 infection. Lancet Infect Dis. (2020). 10.1016/S1473-3099(20)30195-X. [Epub ahead of print]. 32171390 PMC7118515

[3] Zhang YP Analysis of Epidemiological characteristics of new coronavirus pneumonia. Chin. J. Epidemiol. (2020) 41:1–7. 10.3760/cma.j.issn.0254-6450.2020.02.003

[4] Korean Centers for Disease Control and Prevention The Updates on COVID-19 in Korea as of 29 April. (2020). Available online at: https://www.cdc.go.kr/board/board.es?mid=a30402000000&bid=0030

[5] Statistics Korea 2018 Population and Housing Census (2018).

[6] National Bureau of Statistics of China China Statistical Yearbook. (2018). Available online at: http://www.stats.gov.cn/tjsj/ndsj/2018/indexeh.htm

[7] Yuan S China: Wuhan revises coronavirus death toll up by 50 percent. Aljazeera. (2020, April 17). Available online at: https://www.aljazeera.com/news/2020/04/china-wuhan-revises-coronavirus-death-toll-50-200417111645417.html

[8] Burn-Murdoch J Romei V Giles C Global coronavirus death toll could be 60% higher than reported. The Financial Times. (2020, April 26). Available online at: https://www.ft.com/content/6bd88b7d-3386-4543-b2e9-0d5c6fac846c

[9] The Economist Tracking covid-19 excess deaths across countries (2020, April 16). Available online at: https://www.economist.com/graphic-detail/2020/04/16/tracking-covid-19-excess-deaths-across-countries?utm_campaign=coronavirus-special-edition&utm_medium=newsletter&utm_source=salesforce-marketing-cloud&utm_term=2020-04-25&utm_content=article-link-6

[10] Reichert TA Simonsen L Sharma A Pardo SA Fedson D Miller MA. Influenza and the winter increase in mortality in the United States, 1959–1999. Am J Epidemiol. (2004) 160:492–502. 10.1093/aje/kwh227 15321847

[11] Simonsen L Clarke MJ Schonberger LB Arden NH Cox NJ Fukuda K. Pandemic versus epidemic influenza mortality: a pattern of changing age distribution. J Infect Dis. (1998)178:53–60. 10.1086/515616 9652423

[12] Lemaitre M Carrat F. Comparative age distribution of influenza morbidity and mortality during seasonal influenza epidemics and the 2009 H1N1 pandemic. BMC Infect Dis. (2010). 10:162. 10.1186/1471-2334-10-162 20534113 PMC2896934

[13] Eurostat EU Cities – The Young and the Old. (2019). Available online at: https://ec.europa.eu/eurostat/web/products-eurostat-news/-/EDN-20191030-1

[14] The World Bank Age Dependency Ratio, Old (% of Working-Age Population). (n.d.). Available online at: https://data.worldbank.org/indicator/SP.POP.DPND.OL?most_recent_value_desc=true

